# Debut of Gastroesophageal Reflux Concomitant with Administration of Sublingual Immunotherapy

**DOI:** 10.1155/2017/8905372

**Published:** 2017-10-04

**Authors:** Jacob Juel

**Affiliations:** Department of Plastic Surgery, Aalborg University Hospital, Aalborg, Denmark

## Abstract

Gastroesophageal reflux disease (GORD) is an often debilitating condition characterised by retrograde flow of content from stomach into the oesophagus, where the low pH of the stomach acid irritates the mucosa of the oesophagus. The most dominant symptoms in GORD are pyrosis, regurgitation, and dysphagia. Sublingual immunotherapy (SLIT) was first described in 1986. Following this description, the use has greatly increased in the treatment of allergic rhinitis, as an alternative to subcutaneously administered immunotherapy. Side effects are commonly of oropharyngeal and gastrointestinal nature, for example, swelling, itching, irritation, ulceration of the oropharynx and nausea, abdominal pain, vomiting, and diarrhoea. More serious side effects are dominated by respiratory tract and systemic manifestations. A 30-year-old male experienced refractory, relentless, and debilitation GORD subsequent to administration of sublingual immunotherapy for house dust mite in allergic rhinitis. The patient had to stop the SLIT after two weeks of administration due to GORD. The cessation resulted in rapid resolution of symptoms.

## 1. Introduction

Allergen immunotherapy has traditionally been administered by subcutaneous injections, but regular injections for a period of 3–5 years are often required for efficacy. Conversely, sublingual immunotherapy (SLIT) first described in 1986 [1] is given as a tablet or a liquid extract. Since then, its use has greatly increased globally, representing an attractive alternative to subcutaneously administered immunotherapy in the treatment of allergic rhinitis, as self-administration is much less intricate. The safety profile of SLIT is superior to that of subcutaneously administered immunotherapy, as side effects are less severe and frequent [2]. The clinical efficacy in allergic rhinitis due to house dust mite (HDM) allergens is well-established [3, 4]. Mild side effects are frequent primarily in the initial period of SLIT treatment and include both local and systemic manifestations. Anaphylaxis has been reported but is considered extremely rare [5]. The most common local side effects are of oropharyngeal and gastrointestinal nature, for example, itching, swelling, irritation, and ulceration of the oropharynx and nausea, abdominal pain, diarrhoea, and vomiting, although pyrosis or gastroesophageal reflux has never been described before. Systemic side effects are dominated by respiratory tract manifestations, particularly rhinitis and asthma, but angioedema and urticaria may occur, although less frequently compared to local side effects.

## 2. Case

A 30-year-old male with no history of gastroesophageal disease and with known allergic rhinitis due to HDM (*Dermatophagoides pteronyssinus* specific IgE 11 × 10^3^ IU/L) diagnosed using a radioallergosorbent test, and asthma in childhood, received the first tablet (ACARIZAX 12 SQ-HDM,* Dermatophagoides pteronyssinus* and* Dermatophagoides farinae* 50%/50% ALK-Abelló, Hørsholm, Denmark) at an outpatient facility. The SLIT dosage-regimen was initiated as one tablet a day for a planned three years. The dose was held sublingually for one minute before any swallowing was permitted. The treatment was performed in the morning. During the first few minutes only discrete oropharyngeal itching was present. After 30 minutes the itching had subsided; however, concomitantly a debilitating reflux of stomach acid started. The symptoms included severe pyrosis, regurgitation, and nausea, which subsided a little during the following hours but worsened in relation to any meals and ingestions of any type of liquid. The patient continued on the medication at the prescribed dose in spite of these side effects. While recumbent or supine and during the night, the gastroesophageal reflux intensified, hence the symptoms of pyrosis and regurgitation. On these grounds the patient consulted his general practitioner (GP) after five days. He was prescribed 40 mg of esomeprazole daily. Furthermore, the GP added ranitidine 150 mg before bedtime. The treatment did, however, not resolve the problem and the pyrosis and regurgitation continued relentlessly at the same intensity. After two weeks of treatment the patient decided to stop the SLIT treatment after consulting the prescribing allergologist. The day after the termination of the SLIT, the patient was symptom-free. After six months, no further episodes of symptomatic gastroesophageal reflux have been reported. The SLIT therapy has been abandoned and the patient continues on subcutaneously administered immunotherapy (100,000 SQ-U Alutard® SQ,, ALK-Abelló, Hørsholm, Denmark) with good effect. As the symptoms went away concomitantly with the termination of SLIT, no further investigations were carried out, for example, gastroscopy, 24-hour pH measurement with impedance, and high-resolution oesophageal manometry.

## 3. Discussion

Gastroesophageal reflux is a debilitating symptom and may be classified as GORD if the extent of the symptoms is high enough. The most dominant symptoms are pyrosis, dysphagia, and regurgitation [6]. In the present case, a previously healthy young male with no history of gastrointestinal disease or concomitant medical treatment experienced vigorous and refractory gastroesophageal reflux with debut coincidently with administration of SLIT. The patient only had a history of childhood asthma; however, after the age of eight, he never received treatment. Esomeprazole combined with ranitidine did not yield any amelioration, and only termination of SLIT resolved the problem.

Gastroesophageal reflux is to some extent considered a physiological process, as it often occurs transiently in the postprandial state. Thus, most people experience intermittent or transient gastroesophageal reflux; however, only a smaller proportion fulfils the criteria for diagnosis; hence, in a predominantly Western population the prevalence is found to be between 10 and 20 percent [7].

The most common risk factors of gastroesophageal reflux are hiatus hernia, smoking, certain types of food (i.e., high in saturated fat) and eating pattern, and obesity [8]. It should be also noted that patients with asthma are at higher risk of developing GORD; on the other hand microaspiration in GORD may exacerbate asthma [9]. In this case, the patient did not have any chronic cough or asthmatic symptoms. Medical treatment may as well cause or contribute to gastroesophageal reflux, for example, NSAIDs and muscle relaxants. In the present case, no history of GORD or reflux was present; furthermore, the patient did not receive any medication. In terms of body composition, the patient had a BMI under 25, was a teetotaller, and did not smoke. Thus, there is no evidence of any potential causes of the GORD, and as paralleled in the case with debut, coincidently with the administration of SLIT and the resolution just after the termination of treatment, it must be considered a potential side effect to SLIT. After this episode, no further trials of SLIT have been tried, and the patient was shifted to subcutaneously administered immunotherapy with self-reported satisfactory effect. There were no changes in daily intake of food, routines, or types of food during the SLIT treatment.

An alternative and perhaps more plausible explanation of the refractory symptoms of primarily pyrosis in this patient could be eosinophilic esophagitis (EO), that is, an immune system disease that is characterised by persistent heartburn, upper abdominal pain, and regurgitation refractory to medication, for example, proton pump inhibitors and antacids [10]. Furthermore, as the patient was known to have suffered from childhood asthma, the diagnosis of EO is more likely, as this is a risk factor of EO. The diagnosis of EO is made by clinical symptoms, suboptimal response to gastrooesophageal medications, and mucosal eosinophilic infiltration of oesophagus on biopsies. The general risk factors for EO include interalia allergic respiratory diseases. The primary treatment of EO is topical steroids; however, in the present case, the termination of SLIT would have sufficed, as SLIT previously has been linked to EO [11].

In relation to SLIT, no previous reports of GORD as side effect have been identified; hence, this is the first report per se; although the symptoms may be attributed to EO, a diagnosis of EO cannot be made retrospectively. The low incidents of GORD or GORD because of EO may be due to GORD being a frequently reported symptom in any population and therefore not regarded as a problem by the individual, underreporting, or very low incidence of GORD due to SLIT.

A short escalation in dose could potentially circumvent the initial side effects; AZARIZAX is, however, only available in 12 SQ-HDM, but other SLIT products are also available. Taken together, the pathophysiological mechanisms of monosymptomatic GORD induced by SLIT with HDM allergen remain unknown, as none of the enumerated causes fit the current case. Relaxation of the lower oesophageal sphincter because of mild allergic/asthmatic reaction due to SLIT allergens may provide a theoretical explanation.

## 4. Conclusion

The details of this case provide important insights for clinical practice, as GORD has not previously been reported as a side effect of SLIT with HDM allergens. Although side effects may occur, SLIT remains safe and efficacious. Informing patients receiving SLIT remains important when prescribing SLIT and should include information regarding risks of both local and systemic side effects including GORD and the risk of change of treatment from SLIT to, for example, subcutaneously administered immunotherapy, due to side effects such as GORD. Contrarily, if a diagnosis of EO is more probable, the treatment protocol of this disease should be followed.

## References

[1] Scadding G. K., Brostoff J. (1986). Low dose sublingual therapy in patients with allergic rhinitis due to house dust mite. *Clinical & Experimental Allergy*.

[2] Cox L. S., Linnemann D. L., Nolte H., Weldon D., Finegold I., Nelson H. S. (2006). Sublingual immunotherapy: a comprehensive review. *Journal of Allergy and Clinical Immunology*.

[3] Compalati E., Passalacqua G., Bonini M., Canonica G. W. (2009). The efficacy of sublingual immunotherapy for house dust mites respiratory allergy: Results of a GALEN meta-analysis. *Allergy: European Journal of Allergy and Clinical Immunology*.

[4] Jin J. J., Li J. T., Klimek L., Pfaar O. (2017). Sublingual Immunotherapy Dosing Regimens: What Is Ideal?. *Journal of Allergy and Clinical Immunology: In Practice*.

[5] Van Dyken A. M., Smith P. K., Fox T. L. (2014). Clinical case of anaphylaxis with sublingual immunotherapy: House dust mite allergen. *Journal of Allergy and Clinical Immunology: In Practice*.

[6] Kahrilas P. J. (2008). Gastroesophageal reflux disease. *The New England Journal of Medicine*.

[7] Dent J., El-Serag H. B., Wallander M.-A., Johansson S. (2005). Epidemiology of gastro-oesophageal reflux disease: a systematic review. *Gut*.

[8] Khan A. (2016). Impact of obesity treatment on gastroesophageal reflux disease. *World Journal of Gastroenterology*.

[9] Ciorba A. (2015). Upper aerodigestive tract disorders and gastro-oesophageal reflux disease. *World Journal of Clinical Cases*.

[10] Dellon E. S., Hirano I. (2017). Epidemiology and Natural History of Eosinophilic Esophagitis. *Gastroenterology*.

[11] Béné J., Ley D., Roboubi R., Gottrand F., Gautier S. (2016). Eosinophilic esophagitis after desensitization to dust mites with sublingual immunotherapy. *Annals of Allergy, Asthma and Immunology*.

